# Synthesis and structure of 1,1-bis­(furan-2-ylmethyl)-3-phenyl­urea

**DOI:** 10.1107/S2056989026010029

**Published:** 2026-10-02

**Authors:** Dimitriy F. Mertsalov, Dimitriy M. Schevnikov, Victor N. Khrustalev, Tuncer Hökelek, Alebel N. Belay, Khudayar I. Hasanov, Ibadulla H. Mahmudov

**Affiliations:** a RUDN University, 6 Miklukho-Maklaya St., Moscow 117198, Russian Federation; b Zelinsky Institute of Organic Chemistry of RAS, Leninsky Prospect 47, Moscow 119991, Russian Federation; c Hacettepe University, Department of Physics, 06800 Beytepe-Ankara, Türkiye; d Department of Chemistry, Bahir Dar University, PO Box 79, Bahir Dar, Ethiopia; e Azerbaijan Medical University, Scientific Research Centre (SRC), A. Kasumzade Str. 14, AZ 1022, Baku, Azerbaijan; f Department of Chemical Engineering, Baku Engineering University, Hasan Aliyev Str. 120, AZ0101, Khirdalan, Absheron, Azerbaijan; University of Aberdeen, United Kingdom

**Keywords:** furan, crystal structure, non-covalent inter­actions

## Abstract

The asymmetric unit of the title compound, C_17_H_16_N_2_O_3_, contains two mol­ecules, *A* and *B*, with one furan ring in each mol­ecule showing ‘flip’ rotational disorder. In the crystal, N—H⋯O hydrogen bonds link the mol­ecules into separate infinite ⋯*A*⋯*A*⋯*A*⋯ and ⋯*B*⋯*B*⋯*B*⋯ chains propagating along the *c*-axis direction. Weak C—H⋯π inter­actions may help to consolidate the packing. Hirshfeld surface analysis revealed that the most important contributions to the packing are from H⋯H, H⋯C/C⋯H and H⋯O/O⋯H inter­actions with very similar surface percentages for both mol­ecules.

## Abstract 2

The asymmetric unit of the title compound contains two mol­ecules, *A* and *B*, with one furan ring in each mol­ecule showing ‘flip’ rotational disorder. In the crystal, N—H⋯O hydrogen bonds link the mol­ecules into separate infinite ⋯*A*⋯*A*⋯*A*⋯ and ⋯*B*⋯*B*⋯*B*⋯ chains propagating along the *c*-axis direction.

## 1. Chemical context

Difurfuryl­amine, C_10_H_11_NO_2_ (DiFAM), is a common precursor chemical with potential applications in pharmaceuticals (Zhuang *et al.*, 2022 ▸). Particular inter­est is invested into the use of DiFAM in the synthesis of di­thio­carbamates: such compounds exhibit anti­tumor activity, are used as catalysts and biocides (Tamilvanan *et al.*, 2017 ▸), and thallium(III) *N*,*N*-difurfuryldi­thio­carbamate has an oxidative property towards organic mol­ecules, is used within anti-alcohol medicine and possesses significant fungicidial activity (Gomathi *et al.*, 2023 ▸). Ureas themselves have shown a wide variety of applications and their anti­microbial (Resende *et al.*, 2023 ▸) and anti­bacterial (Yang *et al.*, 2025 ▸) activities have led to applications such as soporifics and local and general anaesthetics or herbicides (fenuron, isoproturon, sulfentrazone) and pesticides (diuron, monolinuron, chloro­toluron). The combination of the urea skeleton with a pharmaceutically promising difurfuryl­amine structure may lead to a new library of biologically active compounds. Inter­est in difurfuryl­amine chemistry was sparked in us in 2019, when difurfurylethers, alkenes and amides showed good selectivity in reactions with electron-deficient dimethyl acetyl­enedi­carboxyl­ate, producing kinetically and thermodynamically controlled di­epoxy­naphthalenes (Borisova *et al.*, 2018 ▸). We decided to introduce different substituents to the nitro­gen atom of difurfuryl­amine, in this case transforming it into the title compound, C_17_H_16_N_2_O_3_ (**3**) and we now describe its structure.
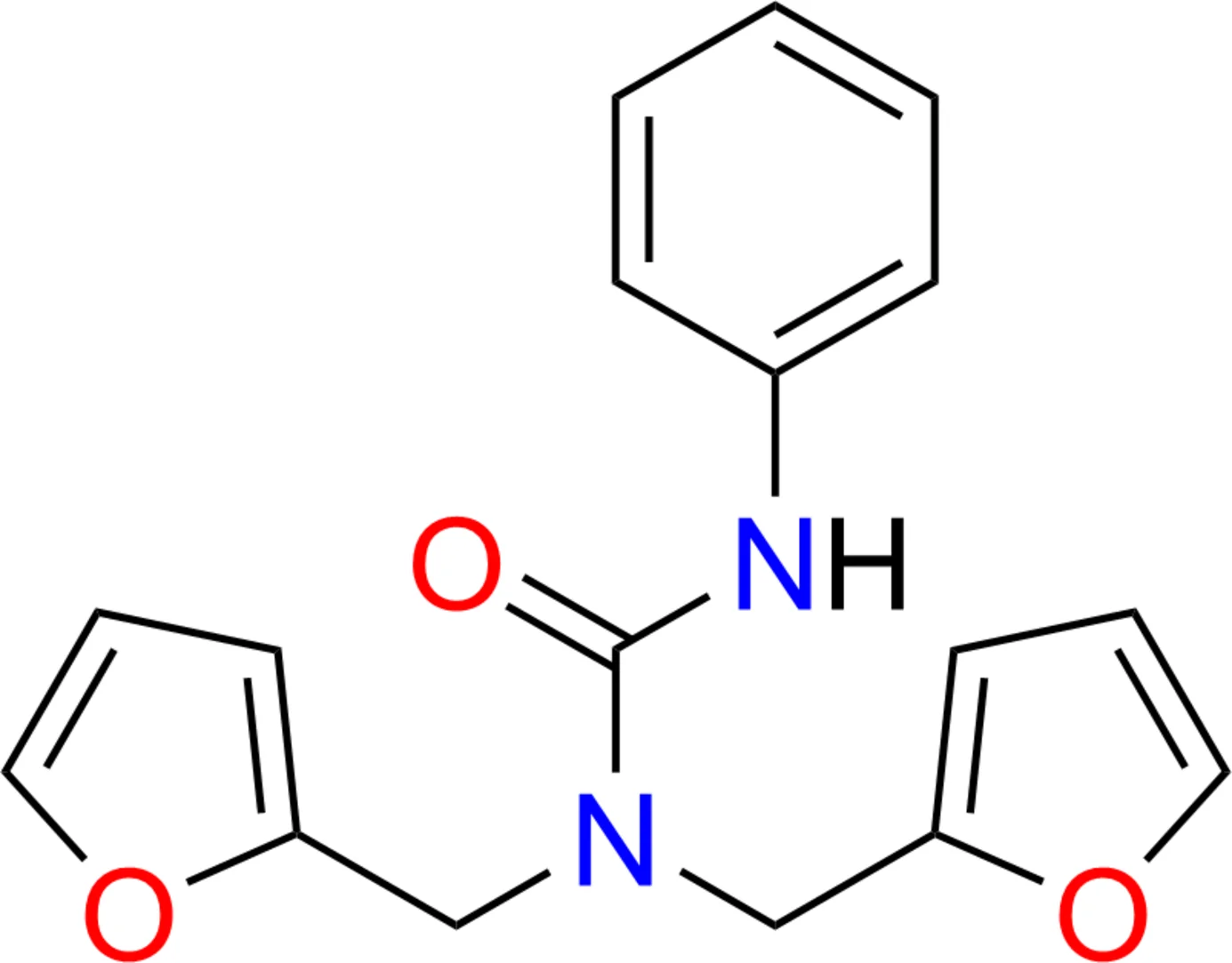


## 2. Structural commentary

The asymmetric unit of (**3**) contains two mol­ecules, *A* and *B* (Fig. 1 ▸). In each mol­ecule, one furan ring is well ordered, while the other [the furan *B* ring (O3/C8–C11) in *A* and the *D* ring (O5/C20–C23) in *B*] is disordered over two sets of sites related by a ‘flip’ (∼180°) rotation about the C—C bond connecting it to the adjacent methyl­ene group. In mol­ecule *A*, the ordered furan, *A* (O2/C3–C6) and phenyl, *C* (C12–C17) rings are oriented at a dihedral angle of *A*/*C* = 87.60 (19)°. In mol­ecule *B*, the furan, *E* (O6/C25–C28) and phenyl, *F* (C29–C34) rings are oriented at a dihedral angle of *E*/*F* = 74.12 (17)°. There are no significant differences between the bond distances in *A* and *B* but the C7—N1—C1 [121.20 (19)°], C1—N1—C2 [115.52 (19)°], O1—C1—N2 [123.1 (2)°] and C1—N1—C12 [125.8 (2)°] bond angles in *A* are slightly different from the corresponding C18—N3—C24 [123.74 (19)°], C19—N3—C18 [118.2 (2)°], O4—C18—N4 [122.2 (2)°] and C18—N4—C29 [124.1 (2)°] bond angles in *B*.

## 3. Supra­molecular features

In the crystal, N—H⋯O hydrogen bonds (Table 1 ▸) link the mol­ecules into separate infinite ⋯*A*⋯*A*⋯*A*⋯ and ⋯*B*⋯*B*⋯*B*⋯ *C*(4) chains propagating along the *c*-axis direction (Fig. 2 ▸). Adjacent mol­ecules are generated by *c*-glide symmetry in each case. Weak C—H⋯π inter­actions may help to consolidate the packing.

## 4. Hirshfeld surface analysis

For visualizing the inter­molecular inter­actions in the crystal of (**3**), Hirshfeld surface (HS) analyses were carried out using *Crystal Explorer 17.5* (Spackman *et al.*, 2021 ▸). Only the major disorder components of furan rings were taken into account for the analysis. In the HSs plotted over *d*_norm_ (Fig. 3 ▸*a*,*b*), the red spots indicate their roles as the respective donors and/or acceptors in hydrogen bonding, as discussed above. The overall two-dimensional fingerprint plots are shown in Fig. 4 ▸*a* and 5*a* and those delineated into the different contact types are illustrated in Fig. 4 ▸(*b*)–(*j*) and Fig. 5 ▸(*b*)–(*j*) for mol­ecules *A* and *B*, respectively. Their contributions to the HSs are presented in Table 2 ▸. Comparison of their percentages shows that there are no significant differences, implying that their environments in the crystal are very similar.

## 5. Synthesis and crystallization

Bis(furan-2-yl)amine (**1**) (100 mg, 0.56 mmol) was dissolved in benzene (5 ml) at room temperature (Fig. 6 ▸). Iso­cyanato­benzene (**2**) (74 mg, 0.62 mmol) was added to the solution and the reaction was refluxed for 6 h (progress monitored by TLC). The resulting mixture was cooled, and the formation of a solid was observed. The crystals were filtered off, washed with diethyl ether (3 × 5 ml), dried under vacuum, and then air. The target compound (**3**) did not require further purification; yield 70%, 117.1 mg, colourless crystals, m.p. 381–383 K. Single crystals of (**3**) were grown from a solvent mixture of ethanol and *N*,*N*-di­methyl­formamide. IR (KBr), ν (cm^−1^): 1725, ^1^H NMR (700 MHz, DMSO-*d*_6_, 273 K) (*J* Hz): δ 8.57 (*s*, 1H), 7.60 (*dd*, *J* = 0.9, 1.9 Hz, 2H), 7.47 (*d*, *J* = 7.6 Hz, 2H), 7.24 (*t*, *J* = 7.6 Hz, 2H), 6.96 (*t*, *J* = 7.6 Hz, 1H), 6.40 (*dd*, *J* = 1.9, 3.10 Hz, 2H), 6.31 (*d*, *J* = 3.10 Hz, 2H), 4.54 (*s*, 4H) ppm. ^13^C {^1^H} NMR (150.9 MHz, DMSO-*d*_6_, 273 K): δ 155.17, 151.67 (2C), 143.12 (2C), 140.66, 128.79 (2C), 122.53, 120.35 (2C), 110.95 (2C), 108.55 (2C), 42.72 (2C) ppm. MS (ESI) *m*/*z*: 296 [*M* + H]^+^.

## 6. Refinement

Crystal data, data collection and structure refinement details are summarized in Table 3 ▸. The hydrogen atom positions were calculated geometrically at distances of N—H = 0.90 and C—H = 0.95–0.99 Å and refined using a riding model while applying the constraint *U*_iso_(H) = 1.2 × *U*_eq_ C, N). One furan ring in each mol­ecule was found to be disordered over two sets of sites and they were refined with site occupancy ratios of 0.6:0.4 in each case.

## Supplementary Material

Crystal structure: contains datablock(s) I, global. DOI: 10.1107/S2056989026010029/hb8256sup1.cif

Structure factors: contains datablock(s) I. DOI: 10.1107/S2056989026010029/hb8256Isup2.hkl

Supporting information file. DOI: 10.1107/S2056989026010029/hb8256Isup3.cml

CCDC reference: 2591499

Additional supporting information:  crystallographic information; 3D view; checkCIF report

## Figures and Tables

**Figure 1:**
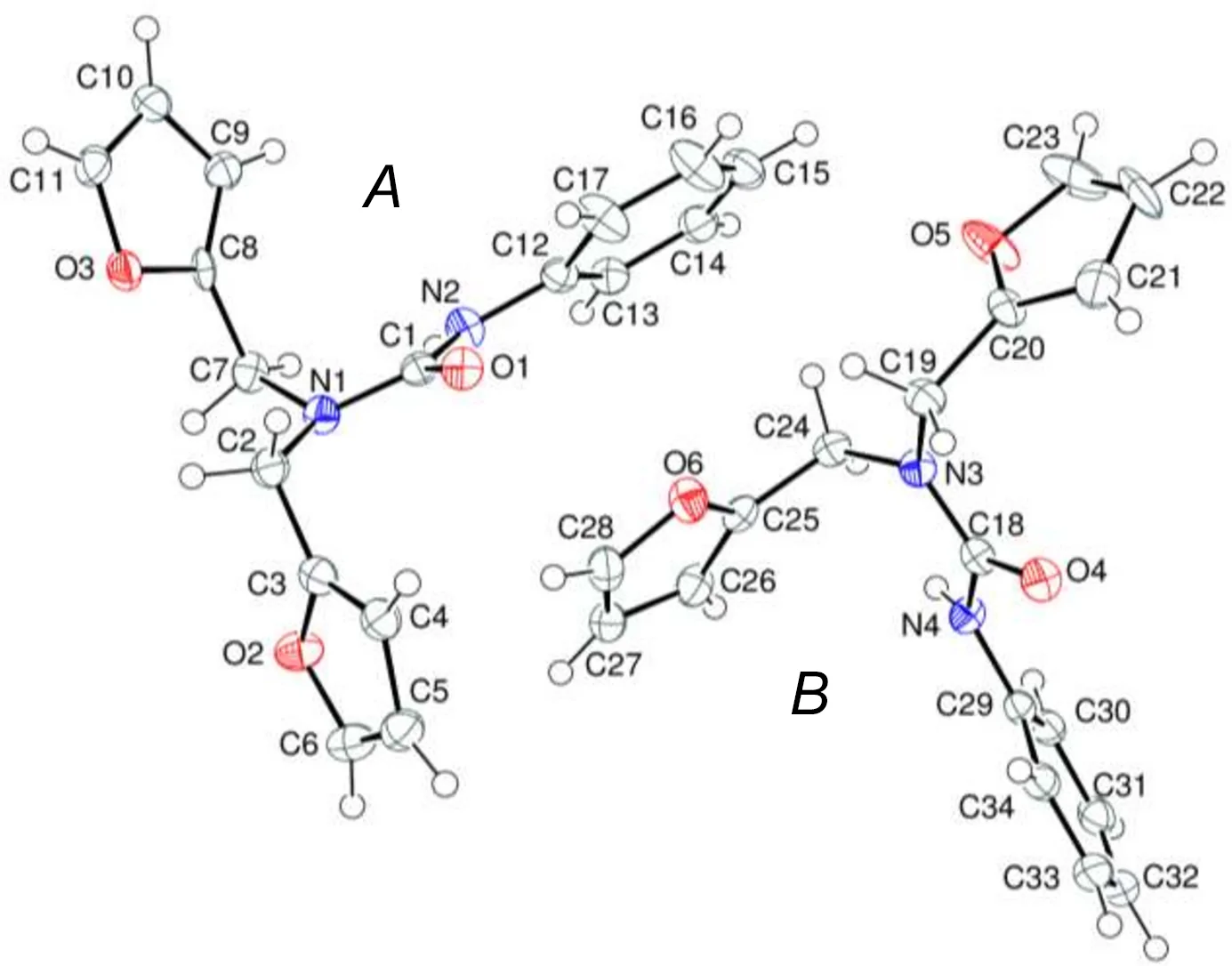
The asymmetric unit of (**3**) with 50% probability ellipsoids. Only the major disorder components of the disordered furan rings are shown.

**Figure 2:**
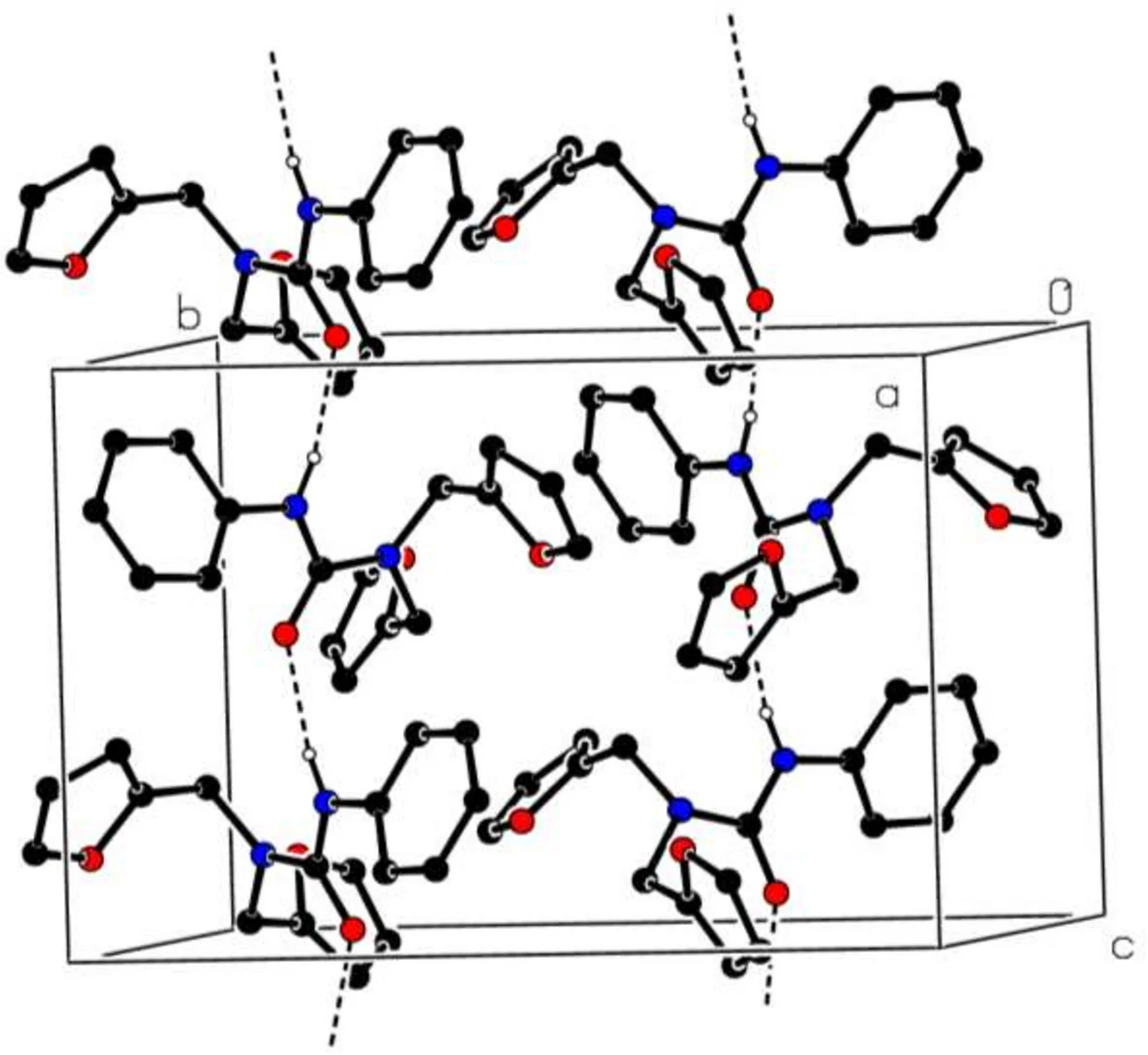
A partial packing diagram of (**3**) with N—H⋯O hydrogen bonds shown as dashed lines. Non-bonding H atoms have been omitted for clarity.

**Figure 3:**
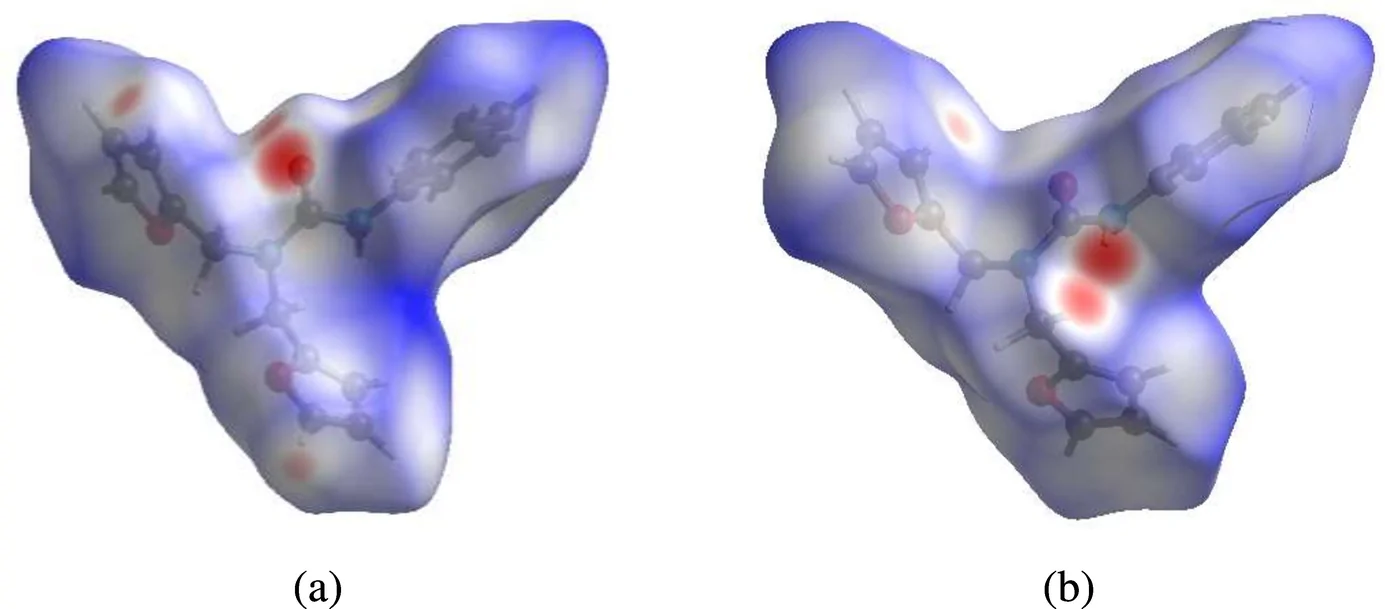
Views of the three-dimensional Hirshfeld surfaces for mol­ecules (*a*) *A* and (*b*) *B* plotted over *d*_norm_ in the ranges from −0.49 to 1.58 a.u. and −0.53 to 1.56 a.u., respectively.

**Figure 4:**
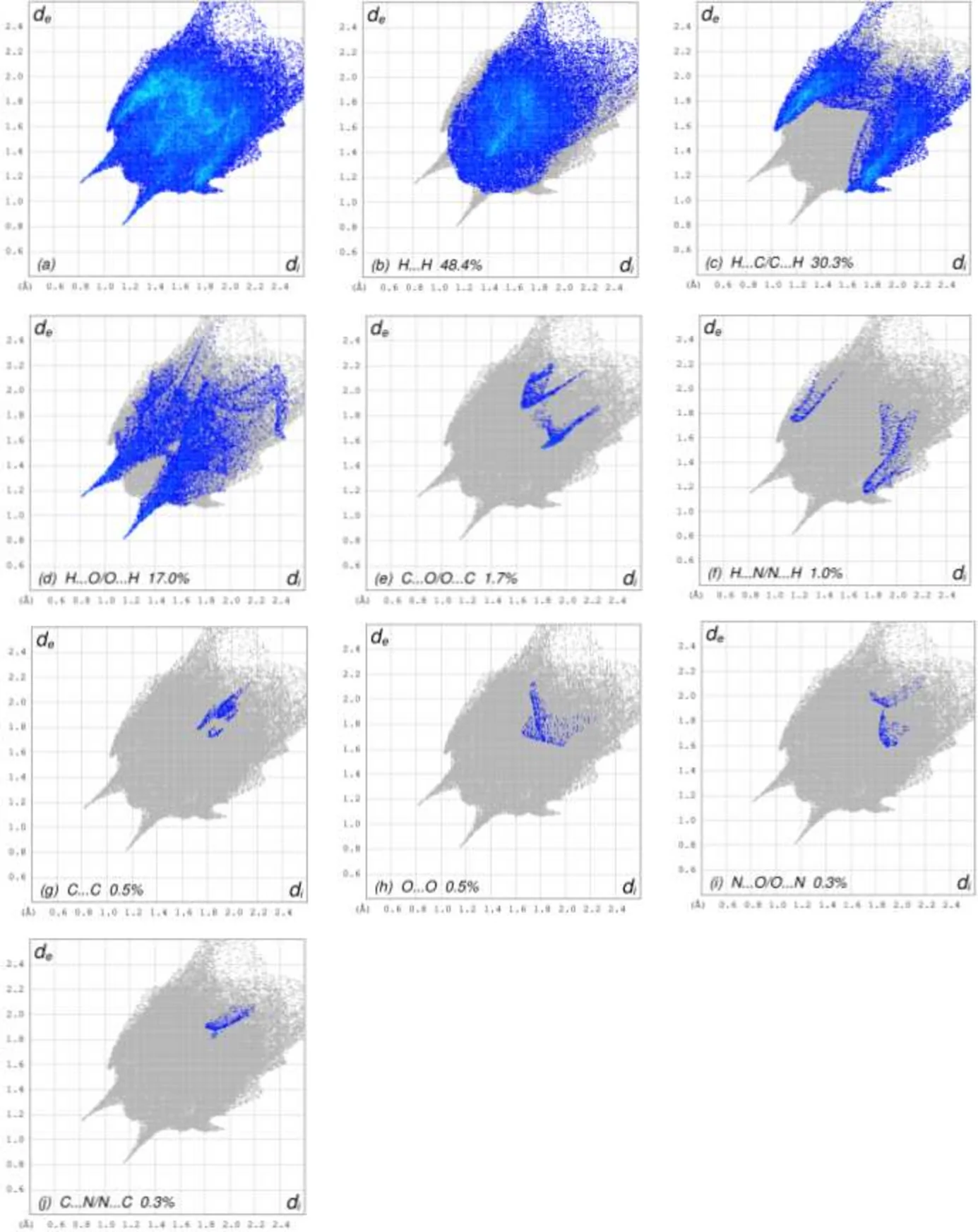
The full two-dimensional fingerprint plots for mol­ecule *A*, showing all inter­actions, and delineated into different contact types.

**Figure 5:**
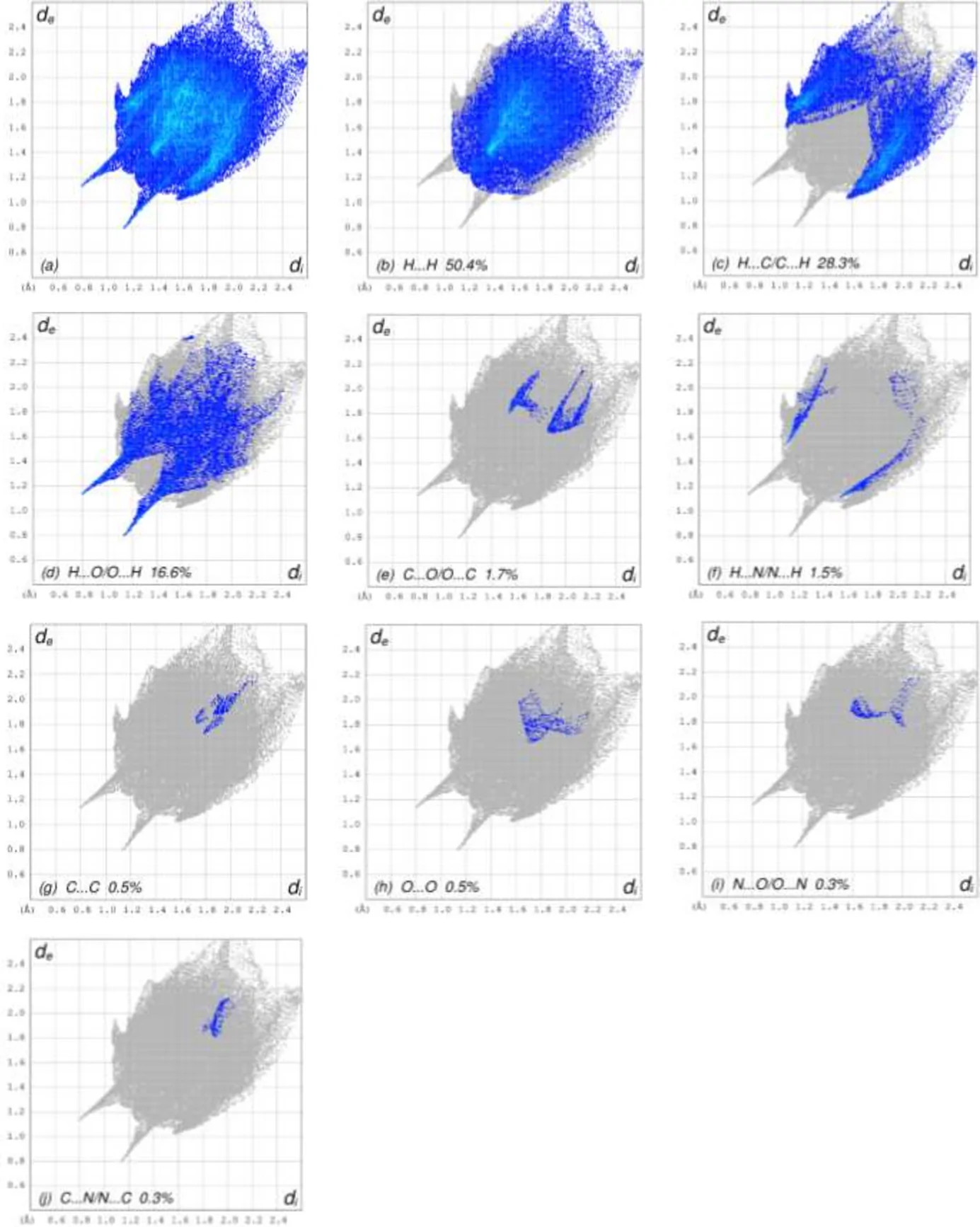
The full two-dimensional fingerprint plots for mol­ecule *B*, showing all inter­actions, and delineated into different contact types.

**Figure 6:**
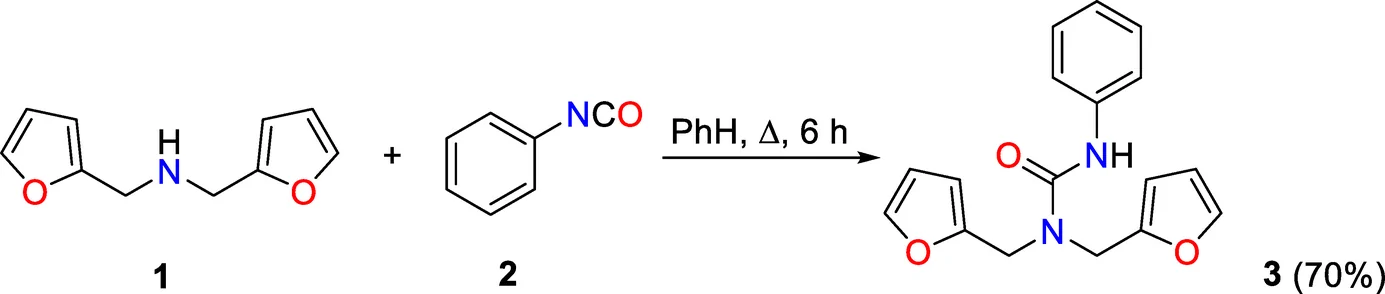
Reaction scheme for obtaining (**3**).

**Table 1:** Hydrogen-bond geometry (Å, °) *Cg*1, *Cg*5 and *Cg*6 are the centroids of the O2/C3–C6, C12–C17 and C29–C34 rings, respectively.

*D*—H⋯*A*	*D*—H	H⋯*A*	*D*⋯*A*	*D*—H⋯*A*
N2—H2*N*⋯O1^i^	0.90	2.03	2.917 (2)	168
N4—H4*N*⋯O4^ii^	0.90	2.07	2.955 (2)	167
C6—H6⋯*Cg*6^iii^	0.95	2.62	3.482	150
C24—H24*B*⋯*Cg*5^iv^	0.99	2.90	3.789	149
C28—H28⋯*Cg*1^iv^	0.95	2.82	3.658	148

Symmetry codes: (i) 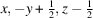; (ii) 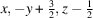; (iii) 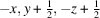; (iv) 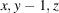.

**Table 2:** Comparison of the Hirshfeld fingerprint percentages for mol­ecules *A* and *B*

Contact	*A*	*B*
H⋯H	48.4	50.4
H⋯C/C⋯H	30.3	28.3
H⋯O/O⋯H	17.0	16.6
C⋯O/O⋯C	1.7	1.7
H⋯N/N⋯H	1.0	1.5
C⋯C	0.5	0.5
O⋯O	0.5	0.5
O⋯N/N⋯O	0.3	0.3
C⋯N/N⋯C	0.3	0.3

**Table 3:** Experimental details

Crystal data	
Chemical formula	C_17_H_16_N_2_O_3_
*M*_r_	296.32
Crystal system, space group	Monoclinic, *P*2_1_/*c*
Temperature (K)	100
*a*, *b*, *c* (Å)	20.1286 (2), 14.76143 (13), 9.97556 (8)
β (°)	92.8316 (8)
*V* (Å^3^)	2960.39 (5)
*Z*	8
Radiation type	Cu *K*α
μ (mm^−1^)	0.76
Crystal size (mm)	0.39 × 0.15 × 0.09

Data collection	
Diffractometer	Micro-focus sealed X-ray tube
Absorption correction	Gaussian (*CrysAlis PRO*; Rigaku OD, 2024 ▸)
*T*_min_, *T*_max_	0.708, 1.000
No. of measured, independent and observed [*I* > 2σ(*I*)] reflections	34327, 6425, 5840
*R*_int_	0.039
(sin θ/λ)_max_ (Å^−1^)	0.639

Refinement	
*R*[*F*^2^ > 2σ(*F*^2^)], *wR*(*F*^2^), *S*	0.068, 0.181, 1.09
No. of reflections	6425
No. of parameters	439
No. of restraints	20
H-atom treatment	H-atom parameters constrained
Δρ_max_, Δρ_min_ (e Å^−3^)	0.59, −0.50

Computer programs: *CrysAlis PRO* (Rigaku OD, 2024 ▸), *SHELXT* (Sheldrick, 2015*a* ▸), *SHELXL* (Sheldrick, 2015*b* ▸) and *SHELXTL* (Sheldrick, 2008 ▸).

## supplementary crystallographic information

### 1,1-Bis(furan-2-ylmethyl)-3-phenylurea Crystal data

**Table:**

C_17_H_16_N_2_O_3_	*F*(000) = 1248
*M**_r_* = 296.32	*D*_x_ = 1.330 Mg m^−^^3^
Monoclinic, *P*2_1_/*c*	Cu *K*α radiation, λ = 1.54184 Å
*a* = 20.1286 (2) Å	Cell parameters from 21717 reflections
*b* = 14.76143 (13) Å	θ = 2.3–79.8°
*c* = 9.97556 (8) Å	µ = 0.76 mm^−^^1^
β = 92.8316 (8)°	*T* = 100 K
*V* = 2960.39 (5) Å^3^	Plate, colourless
*Z* = 8	0.39 × 0.15 × 0.09 mm

### 1,1-Bis(furan-2-ylmethyl)-3-phenylurea Data collection

**Table:**

Micro-focus sealed X-ray tube diffractometer	5840 reflections with *I* > 2σ(*I*)
Detector resolution: φ and ω pixels mm^-1^	*R*_int_ = 0.039
Rigaku XtaLAB Synergy–S, HyPix–6000HE area–detector scans	θ_max_ = 79.9°, θ_min_ = 2.2°
Absorption correction: gaussian (CrysAlisPro; Rigaku OD, 2024)	*h* = −25→25
*T*_min_ = 0.708, *T*_max_ = 1.000	*k* = −18→18
34327 measured reflections	*l* = −11→12
6425 independent reflections	

### 1,1-Bis(furan-2-ylmethyl)-3-phenylurea Refinement

**Table:**

Refinement on *F*^2^	Primary atom site location: difference Fourier map
Least-squares matrix: full	Secondary atom site location: difference Fourier map
*R*[*F*^2^ > 2σ(*F*^2^)] = 0.068	Hydrogen site location: mixed
*wR*(*F*^2^) = 0.181	H-atom parameters constrained
*S* = 1.09	*w* = 1/[σ^2^(*F*_o_^2^) + (0.053*P*)^2^ + 5.931*P*] where *P* = (*F*_o_^2^ + 2*F*_c_^2^)/3
6425 reflections	(Δ/σ)_max_ < 0.001
439 parameters	Δρ_max_ = 0.59 e Å^−^^3^
20 restraints	Δρ_min_ = −0.50 e Å^−^^3^

### 1,1-Bis(furan-2-ylmethyl)-3-phenylurea Special details

**Table:**

Geometry. All esds (except the esd in the dihedral angle between two l.s. planes) are estimated using the full covariance matrix. The cell esds are taken into account individually in the estimation of esds in distances, angles and torsion angles; correlations between esds in cell parameters are only used when they are defined by crystal symmetry. An approximate (isotropic) treatment of cell esds is used for estimating esds involving l.s. planes.

### 1,1-Bis(furan-2-ylmethyl)-3-phenylurea Fractional atomic coordinates and isotropic or equivalent isotropic displacement parameters (Å^2^)

**Table:**

	*x*	*y*	*z*	*U*_iso_*/*U*_eq_	Occ. (<1)
O1	0.73597 (8)	0.26382 (12)	0.41633 (16)	0.0238 (4)	
O2	0.93019 (9)	0.19490 (13)	0.33124 (17)	0.0292 (4)	
O3	0.72614 (15)	−0.0280 (2)	0.2890 (4)	0.0258 (7)	0.6
O3'	0.6514 (4)	0.0572 (6)	0.1365 (9)	0.0369 (11)	0.4
N1	0.78252 (10)	0.16531 (14)	0.2722 (2)	0.0228 (4)	
N2	0.70445 (10)	0.27093 (14)	0.19393 (19)	0.0235 (4)	
H2N	0.718467	0.254403	0.113215	0.028*	
C1	0.73968 (11)	0.23500 (15)	0.3017 (2)	0.0199 (4)	
C2	0.82410 (12)	0.13022 (17)	0.3869 (2)	0.0251 (5)	
H2A	0.840890	0.069373	0.364152	0.030*	
H2B	0.796338	0.123743	0.465540	0.030*	
C3	0.88149 (12)	0.19036 (16)	0.4231 (2)	0.0230 (5)	
C4	0.89752 (12)	0.24433 (17)	0.5291 (2)	0.0256 (5)	
H4	0.872377	0.253027	0.606158	0.031*	
C5	0.95961 (13)	0.28578 (18)	0.5027 (3)	0.0291 (5)	
H5	0.983903	0.327312	0.558896	0.035*	
C6	0.97693 (13)	0.25468 (19)	0.3831 (3)	0.0308 (6)	
H6	1.016140	0.271663	0.340117	0.037*	
C7	0.76556 (12)	0.09991 (16)	0.1638 (2)	0.0243 (5)	
H7A	0.805435	0.063271	0.146441	0.029*	
H7B	0.752968	0.133674	0.080602	0.029*	
C8	0.7098 (3)	0.0376 (6)	0.1960 (9)	0.0177 (9)	0.6
C9	0.6447 (3)	0.0330 (4)	0.1597 (7)	0.0289 (13)	0.6
H9A	0.620994	0.071540	0.097844	0.035*	0.6
C10	0.61794 (19)	−0.0410 (3)	0.2322 (5)	0.0270 (10)	0.6
H10A	0.573193	−0.061697	0.227695	0.032*	0.6
C11	0.66879 (19)	−0.0753 (3)	0.3076 (4)	0.0283 (9)	0.6
H11A	0.665511	−0.125728	0.366157	0.034*	0.6
C8'	0.7112 (6)	0.0382 (13)	0.202 (2)	0.0369 (11)	0.4
C9'	0.7046 (4)	−0.0346 (8)	0.2804 (12)	0.0369 (11)	0.4
H9B	0.738754	−0.062110	0.335402	0.044*	0.4
C10'	0.6368 (4)	−0.0636 (6)	0.2663 (10)	0.0369 (11)	0.4
H10B	0.617100	−0.113450	0.309578	0.044*	0.4
C11'	0.6071 (4)	−0.0056 (5)	0.1794 (8)	0.0369 (11)	0.4
H11B	0.561389	−0.007764	0.151164	0.044*	0.4
C12	0.65565 (12)	0.33951 (16)	0.1991 (2)	0.0228 (5)	
C13	0.64293 (12)	0.39180 (17)	0.0844 (2)	0.0257 (5)	
H13	0.667850	0.381825	0.007354	0.031*	
C14	0.59412 (13)	0.45840 (18)	0.0818 (3)	0.0293 (5)	
H14	0.585419	0.492990	0.002518	0.035*	
C15	0.55810 (13)	0.4746 (2)	0.1940 (3)	0.0338 (6)	
H15	0.525088	0.520663	0.192665	0.041*	
C16	0.57075 (15)	0.4232 (2)	0.3076 (3)	0.0422 (7)	
H16	0.546302	0.434362	0.384976	0.051*	
C17	0.61871 (14)	0.3551 (2)	0.3112 (3)	0.0367 (6)	
H17	0.626191	0.319475	0.389759	0.044*	
O4	0.77404 (9)	0.78394 (12)	0.46392 (16)	0.0252 (4)	
O5	0.5736 (2)	0.6855 (4)	0.3492 (5)	0.0407 (13)	0.6
O5'	0.5886 (6)	0.7549 (10)	0.5176 (14)	0.085 (3)	0.4
O6	0.77833 (9)	0.48633 (12)	0.33707 (18)	0.0283 (4)	
N3	0.72207 (10)	0.67406 (14)	0.3382 (2)	0.0224 (4)	
N4	0.80378 (10)	0.76368 (13)	0.24824 (19)	0.0216 (4)	
H4N	0.790312	0.742669	0.166683	0.026*	
C18	0.76737 (11)	0.74267 (16)	0.3562 (2)	0.0202 (4)	
C19	0.68232 (12)	0.64849 (17)	0.4523 (3)	0.0260 (5)	
H18A	0.709067	0.660842	0.536268	0.031*	
H19B	0.674325	0.582352	0.448025	0.031*	
C20	0.6152 (7)	0.6961 (14)	0.4609 (11)	0.0221 (15)	0.6
C21	0.5916 (3)	0.7523 (6)	0.5522 (7)	0.0411 (18)	0.6
H21A	0.613037	0.771586	0.634353	0.049*	0.6
C22	0.5269 (3)	0.7769 (4)	0.4983 (7)	0.048 (2)	0.6
H22A	0.495344	0.814112	0.539855	0.058*	0.6
C23	0.5196 (3)	0.7374 (5)	0.3783 (7)	0.055 (2)	0.6
H23A	0.481364	0.744451	0.319557	0.066*	0.6
C20'	0.6202 (11)	0.693 (2)	0.441 (2)	0.0221 (15)	0.4
C21'	0.5714 (8)	0.6598 (13)	0.359 (2)	0.085 (3)	0.4
H21B	0.577458	0.611488	0.297609	0.102*	0.4
C22'	0.5093 (8)	0.7047 (10)	0.374 (2)	0.085 (3)	0.4
H22B	0.467395	0.692505	0.329415	0.102*	0.4
C23'	0.5250 (7)	0.7681 (13)	0.466 (2)	0.085 (3)	0.4
H23B	0.496192	0.815340	0.491410	0.102*	0.4
C24	0.72054 (12)	0.61143 (16)	0.2234 (2)	0.0235 (5)	
H24A	0.722451	0.646887	0.139457	0.028*	
H24B	0.677921	0.577832	0.220027	0.028*	
C25	0.77646 (12)	0.54547 (15)	0.2306 (2)	0.0222 (5)	
C26	0.82809 (13)	0.53027 (17)	0.1531 (3)	0.0287 (5)	
H26	0.838036	0.561857	0.073551	0.034*	
C27	0.86519 (13)	0.45689 (18)	0.2143 (3)	0.0300 (5)	
H27	0.904671	0.430420	0.183513	0.036*	
C28	0.83324 (13)	0.43317 (18)	0.3230 (3)	0.0294 (5)	
H28	0.846796	0.385737	0.382614	0.035*	
C29	0.85387 (11)	0.83123 (16)	0.2496 (2)	0.0214 (5)	
C30	0.86190 (12)	0.88096 (17)	0.1319 (3)	0.0253 (5)	
H30	0.833586	0.869805	0.054597	0.030*	
C31	0.91132 (13)	0.94669 (17)	0.1280 (3)	0.0297 (5)	
H31	0.916819	0.979950	0.047775	0.036*	
C32	0.95276 (13)	0.96400 (18)	0.2409 (3)	0.0320 (6)	
H32	0.985742	1.009955	0.238964	0.038*	
C33	0.94535 (13)	0.91352 (19)	0.3560 (3)	0.0312 (6)	
H33	0.973921	0.924638	0.432916	0.037*	
C34	0.89680 (12)	0.84679 (17)	0.3611 (3)	0.0260 (5)	
H34	0.892900	0.811896	0.440347	0.031*	

### 1,1-Bis(furan-2-ylmethyl)-3-phenylurea Atomic displacement parameters (Å^2^)

**Table:**

	*U*^11^	*U*^22^	*U*^33^	*U*^12^	*U*^13^	*U*^23^
O1	0.0288 (9)	0.0276 (9)	0.0151 (7)	0.0001 (7)	0.0024 (6)	0.0014 (6)
O2	0.0275 (9)	0.0370 (10)	0.0234 (8)	−0.0056 (8)	0.0036 (7)	0.0004 (7)
O3	0.0258 (17)	0.0215 (16)	0.0304 (16)	0.0044 (14)	0.0038 (16)	0.0046 (13)
O3'	0.032 (2)	0.042 (3)	0.038 (2)	−0.009 (2)	0.0042 (17)	−0.0037 (19)
N1	0.0245 (10)	0.0221 (10)	0.0218 (10)	0.0007 (8)	−0.0001 (8)	0.0000 (8)
N2	0.0293 (10)	0.0281 (10)	0.0133 (8)	0.0035 (8)	0.0018 (7)	−0.0012 (7)
C1	0.0206 (10)	0.0212 (11)	0.0179 (10)	−0.0029 (8)	0.0015 (8)	0.0009 (8)
C2	0.0255 (12)	0.0230 (11)	0.0264 (12)	0.0005 (9)	−0.0017 (9)	0.0052 (9)
C3	0.0230 (11)	0.0249 (11)	0.0210 (11)	0.0022 (9)	0.0014 (9)	0.0069 (9)
C4	0.0266 (12)	0.0259 (12)	0.0242 (11)	0.0019 (9)	−0.0003 (9)	0.0026 (9)
C5	0.0292 (12)	0.0277 (12)	0.0298 (12)	−0.0029 (10)	−0.0051 (10)	0.0038 (10)
C6	0.0254 (12)	0.0360 (14)	0.0307 (13)	−0.0075 (10)	−0.0004 (10)	0.0049 (11)
C7	0.0293 (12)	0.0236 (11)	0.0203 (11)	0.0005 (9)	0.0045 (9)	−0.0015 (9)
C8	0.029 (2)	0.0126 (18)	0.012 (2)	0.0080 (16)	0.0002 (16)	−0.0028 (15)
C9	0.025 (3)	0.023 (3)	0.037 (3)	−0.001 (2)	−0.006 (2)	−0.004 (2)
C10	0.022 (2)	0.026 (3)	0.033 (3)	0.0009 (19)	0.004 (2)	−0.005 (2)
C11	0.030 (2)	0.021 (2)	0.035 (2)	−0.0007 (18)	0.012 (2)	0.0022 (17)
C8'	0.032 (2)	0.042 (3)	0.038 (2)	−0.009 (2)	0.0042 (17)	−0.0037 (19)
C9'	0.032 (2)	0.042 (3)	0.038 (2)	−0.009 (2)	0.0042 (17)	−0.0037 (19)
C10'	0.032 (2)	0.042 (3)	0.038 (2)	−0.009 (2)	0.0042 (17)	−0.0037 (19)
C11'	0.032 (2)	0.042 (3)	0.038 (2)	−0.009 (2)	0.0042 (17)	−0.0037 (19)
C12	0.0212 (11)	0.0260 (11)	0.0210 (11)	−0.0011 (9)	−0.0016 (8)	−0.0026 (9)
C13	0.0256 (12)	0.0278 (12)	0.0239 (11)	−0.0008 (9)	0.0016 (9)	0.0013 (9)
C14	0.0264 (12)	0.0280 (12)	0.0330 (13)	−0.0015 (10)	−0.0028 (10)	0.0052 (10)
C15	0.0243 (12)	0.0366 (14)	0.0401 (15)	0.0064 (11)	−0.0027 (11)	−0.0027 (12)
C16	0.0338 (14)	0.064 (2)	0.0292 (14)	0.0192 (14)	0.0037 (11)	−0.0027 (13)
C17	0.0317 (14)	0.0534 (18)	0.0251 (13)	0.0133 (13)	0.0033 (10)	0.0053 (12)
O4	0.0304 (9)	0.0293 (9)	0.0161 (7)	0.0015 (7)	0.0027 (6)	−0.0009 (6)
O5	0.0256 (17)	0.074 (4)	0.0217 (16)	0.023 (2)	−0.0078 (13)	−0.003 (2)
O5'	0.053 (4)	0.076 (5)	0.123 (7)	0.017 (3)	−0.020 (4)	−0.046 (5)
O6	0.0329 (9)	0.0257 (9)	0.0268 (9)	0.0035 (7)	0.0059 (7)	0.0060 (7)
N3	0.0246 (10)	0.0230 (10)	0.0198 (9)	0.0002 (8)	0.0045 (7)	0.0016 (7)
N4	0.0258 (10)	0.0229 (10)	0.0161 (9)	−0.0023 (8)	0.0019 (7)	−0.0018 (7)
C18	0.0221 (11)	0.0223 (11)	0.0162 (10)	0.0042 (8)	0.0012 (8)	0.0028 (8)
C19	0.0238 (11)	0.0273 (12)	0.0271 (12)	0.0016 (9)	0.0051 (9)	0.0069 (10)
C20	0.024 (2)	0.0258 (17)	0.017 (4)	0.0033 (15)	0.011 (3)	0.003 (3)
C21	0.045 (4)	0.032 (3)	0.049 (4)	−0.009 (3)	0.030 (3)	−0.005 (3)
C22	0.038 (3)	0.030 (3)	0.081 (5)	0.023 (2)	0.038 (3)	0.025 (3)
C23	0.020 (3)	0.079 (6)	0.065 (4)	0.021 (3)	−0.010 (3)	0.017 (5)
C20'	0.024 (2)	0.0258 (17)	0.017 (4)	0.0033 (15)	0.011 (3)	0.003 (3)
C21'	0.053 (4)	0.076 (5)	0.123 (7)	0.017 (3)	−0.020 (4)	−0.046 (5)
C22'	0.053 (4)	0.076 (5)	0.123 (7)	0.017 (3)	−0.020 (4)	−0.046 (5)
C23'	0.053 (4)	0.076 (5)	0.123 (7)	0.017 (3)	−0.020 (4)	−0.046 (5)
C24	0.0237 (11)	0.0239 (11)	0.0227 (11)	−0.0027 (9)	−0.0006 (9)	0.0010 (9)
C25	0.0267 (11)	0.0191 (11)	0.0205 (11)	−0.0047 (9)	0.0001 (9)	0.0015 (8)
C26	0.0348 (13)	0.0250 (12)	0.0269 (12)	−0.0032 (10)	0.0083 (10)	−0.0023 (10)
C27	0.0286 (12)	0.0256 (12)	0.0358 (14)	0.0001 (10)	0.0034 (10)	−0.0074 (10)
C28	0.0340 (13)	0.0234 (12)	0.0304 (13)	0.0044 (10)	−0.0019 (10)	−0.0013 (10)
C29	0.0204 (10)	0.0203 (11)	0.0237 (11)	0.0025 (8)	0.0038 (8)	−0.0017 (9)
C30	0.0223 (11)	0.0242 (11)	0.0295 (12)	0.0025 (9)	0.0022 (9)	0.0029 (9)
C31	0.0263 (12)	0.0244 (12)	0.0390 (14)	0.0024 (10)	0.0071 (10)	0.0060 (10)
C32	0.0234 (12)	0.0237 (12)	0.0492 (16)	−0.0013 (10)	0.0036 (11)	−0.0022 (11)
C33	0.0244 (12)	0.0319 (13)	0.0371 (14)	−0.0006 (10)	0.0000 (10)	−0.0096 (11)
C34	0.0234 (11)	0.0285 (12)	0.0262 (12)	0.0032 (9)	0.0013 (9)	−0.0033 (10)

### 1,1-Bis(furan-2-ylmethyl)-3-phenylurea Geometric parameters (Å, º)

**Table:**

O1—C1	1.226 (3)	O4—C18	1.236 (3)
O2—C6	1.372 (3)	O5—C20	1.369 (3)
O2—C3	1.376 (3)	O5—C23	1.373 (3)
O3—C11	1.370 (3)	O5'—C20'	1.368 (3)
O3—C8	1.371 (3)	O5'—C23'	1.372 (3)
O3'—C11'	1.369 (3)	O6—C28	1.368 (3)
O3'—C8'	1.371 (3)	O6—C25	1.374 (3)
N1—C1	1.383 (3)	N3—C18	1.369 (3)
N1—C7	1.477 (3)	N3—C24	1.471 (3)
N1—C2	1.478 (3)	N3—C19	1.472 (3)
N2—C1	1.366 (3)	N4—C18	1.368 (3)
N2—C12	1.413 (3)	N4—C29	1.418 (3)
N2—H2N	0.8999	N4—H4N	0.8999
C2—C3	1.487 (3)	C19—C20'	1.414 (14)
C2—H2A	0.9900	C19—C20	1.530 (9)
C2—H2B	0.9900	C19—H18A	0.9900
C3—C4	1.350 (4)	C19—H19B	0.9900
C4—C5	1.428 (4)	C20—C21	1.336 (3)
C4—H4	0.9500	C21—C22	1.432 (3)
C5—C6	1.341 (4)	C21—H21A	0.9500
C5—H5	0.9500	C22—C23	1.333 (3)
C6—H6	0.9500	C22—H22A	0.9500
C7—C8'	1.487 (12)	C23—H23A	0.9500
C7—C8	1.499 (7)	C20'—C21'	1.340 (3)
C7—H7A	0.9900	C21'—C22'	1.429 (3)
C7—H7B	0.9900	C21'—H21B	0.9500
C8—C9	1.343 (3)	C22'—C23'	1.338 (3)
C9—C10	1.430 (3)	C22'—H22B	0.9500
C9—H9A	0.9500	C23'—H23B	0.9500
C10—C11	1.339 (3)	C24—C25	1.487 (3)
C10—H10A	0.9500	C24—H24A	0.9900
C11—H11A	0.9500	C24—H24B	0.9900
C8'—C9'	1.341 (3)	C25—C26	1.345 (3)
C9'—C10'	1.429 (3)	C26—C27	1.435 (4)
C9'—H9B	0.9500	C26—H26	0.9500
C10'—C11'	1.339 (3)	C27—C28	1.335 (4)
C10'—H10B	0.9500	C27—H27	0.9500
C11'—H11B	0.9500	C28—H28	0.9500
C12—C17	1.392 (4)	C29—C34	1.394 (3)
C12—C13	1.393 (3)	C29—C30	1.401 (3)
C13—C14	1.389 (4)	C30—C31	1.391 (4)
C13—H13	0.9500	C30—H30	0.9500
C14—C15	1.384 (4)	C31—C32	1.392 (4)
C14—H14	0.9500	C31—H31	0.9500
C15—C16	1.377 (4)	C32—C33	1.383 (4)
C15—H15	0.9500	C32—H32	0.9500
C16—C17	1.393 (4)	C33—C34	1.390 (4)
C16—H16	0.9500	C33—H33	0.9500
C17—H17	0.9500	C34—H34	0.9500
			
C6—O2—C3	106.2 (2)	C20—O5—C23	102.9 (6)
C11—O3—C8	106.0 (4)	C20'—O5'—C23'	109.5 (14)
C11'—O3'—C8'	106.4 (8)	C28—O6—C25	106.06 (19)
C1—N1—C7	121.20 (19)	C18—N3—C24	123.74 (19)
C1—N1—C2	115.52 (19)	C18—N3—C19	118.2 (2)
C7—N1—C2	116.07 (19)	C24—N3—C19	116.70 (19)
C1—N2—C12	125.8 (2)	C18—N4—C29	124.1 (2)
C1—N2—H2N	115.2	C18—N4—H4N	119.0
C12—N2—H2N	118.4	C29—N4—H4N	115.7
O1—C1—N2	123.1 (2)	O4—C18—N4	122.2 (2)
O1—C1—N1	121.6 (2)	O4—C18—N3	121.4 (2)
N2—C1—N1	115.2 (2)	N4—C18—N3	116.3 (2)
N1—C2—C3	112.47 (19)	C20'—C19—N3	109.2 (14)
N1—C2—H2A	109.1	N3—C19—C20	116.2 (8)
C3—C2—H2A	109.1	N3—C19—H18A	108.2
N1—C2—H2B	109.1	C20—C19—H18A	108.2
C3—C2—H2B	109.1	N3—C19—H19B	108.2
H2A—C2—H2B	107.8	C20—C19—H19B	108.2
C4—C3—O2	110.0 (2)	H18A—C19—H19B	107.4
C4—C3—C2	134.0 (2)	C21—C20—O5	113.8 (7)
O2—C3—C2	116.0 (2)	C21—C20—C19	131.9 (6)
C3—C4—C5	106.6 (2)	O5—C20—C19	114.1 (5)
C3—C4—H4	126.7	C20—C21—C22	104.4 (6)
C5—C4—H4	126.7	C20—C21—H21A	127.8
C6—C5—C4	106.6 (2)	C22—C21—H21A	127.8
C6—C5—H5	126.7	C23—C22—C21	106.5 (5)
C4—C5—H5	126.7	C23—C22—H22A	126.7
C5—C6—O2	110.6 (2)	C21—C22—H22A	126.7
C5—C6—H6	124.7	C22—C23—O5	112.4 (5)
O2—C6—H6	124.7	C22—C23—H23A	123.8
N1—C7—C8'	111.0 (11)	O5—C23—H23A	123.8
N1—C7—C8	113.1 (5)	C21'—C20'—O5'	103.9 (16)
N1—C7—H7A	109.0	C21'—C20'—C19	119.8 (14)
C8—C7—H7A	109.0	O5'—C20'—C19	134.3 (11)
N1—C7—H7B	109.0	C20'—C21'—C22'	112.6 (16)
C8—C7—H7B	109.0	C20'—C21'—H21B	123.7
H7A—C7—H7B	107.8	C22'—C21'—H21B	123.7
C9—C8—O3	110.2 (5)	C23'—C22'—C21'	102.6 (15)
C9—C8—C7	134.8 (4)	C23'—C22'—H22B	128.7
O3—C8—C7	114.9 (4)	C21'—C22'—H22B	128.7
C8—C9—C10	106.8 (5)	C22'—C23'—O5'	110.1 (15)
C8—C9—H9A	126.6	C22'—C23'—H23B	124.9
C10—C9—H9A	126.6	O5'—C23'—H23B	124.9
C11—C10—C9	106.1 (4)	N3—C24—C25	112.77 (19)
C11—C10—H10A	127.0	N3—C24—H24A	109.0
C9—C10—H10A	127.0	C25—C24—H24A	109.0
C10—C11—O3	110.9 (3)	N3—C24—H24B	109.0
C10—C11—H11A	124.5	C25—C24—H24B	109.0
O3—C11—H11A	124.5	H24A—C24—H24B	107.8
C9'—C8'—O3'	109.3 (10)	C26—C25—O6	110.3 (2)
C9'—C8'—C7	137.5 (8)	C26—C25—C24	133.3 (2)
O3'—C8'—C7	113.1 (7)	O6—C25—C24	116.4 (2)
C8'—C9'—C10'	107.8 (9)	C25—C26—C27	106.4 (2)
C8'—C9'—H9B	126.1	C25—C26—H26	126.8
C10'—C9'—H9B	126.1	C27—C26—H26	126.8
C11'—C10'—C9'	105.4 (8)	C28—C27—C26	106.4 (2)
C11'—C10'—H10B	127.3	C28—C27—H27	126.8
C9'—C10'—H10B	127.3	C26—C27—H27	126.8
C10'—C11'—O3'	111.1 (8)	C27—C28—O6	110.9 (2)
C10'—C11'—H11B	124.5	C27—C28—H28	124.5
O3'—C11'—H11B	124.5	O6—C28—H28	124.5
C17—C12—C13	118.9 (2)	C34—C29—C30	119.3 (2)
C17—C12—N2	123.2 (2)	C34—C29—N4	122.4 (2)
C13—C12—N2	117.8 (2)	C30—C29—N4	118.2 (2)
C14—C13—C12	120.6 (2)	C31—C30—C29	120.1 (2)
C14—C13—H13	119.7	C31—C30—H30	120.0
C12—C13—H13	119.7	C29—C30—H30	120.0
C15—C14—C13	120.3 (2)	C30—C31—C32	120.4 (2)
C15—C14—H14	119.8	C30—C31—H31	119.8
C13—C14—H14	119.8	C32—C31—H31	119.8
C16—C15—C14	119.1 (3)	C33—C32—C31	119.2 (2)
C16—C15—H15	120.4	C33—C32—H32	120.4
C14—C15—H15	120.4	C31—C32—H32	120.4
C15—C16—C17	121.2 (3)	C32—C33—C34	121.1 (3)
C15—C16—H16	119.4	C32—C33—H33	119.4
C17—C16—H16	119.4	C34—C33—H33	119.4
C12—C17—C16	119.7 (3)	C33—C34—C29	119.8 (2)
C12—C17—H17	120.1	C33—C34—H34	120.1
C16—C17—H17	120.1	C29—C34—H34	120.1
			
C12—N2—C1—O1	−5.6 (4)	C29—N4—C18—O4	2.5 (4)
C12—N2—C1—N1	177.0 (2)	C29—N4—C18—N3	−178.5 (2)
C7—N1—C1—O1	147.7 (2)	C24—N3—C18—O4	−167.8 (2)
C2—N1—C1—O1	−1.4 (3)	C19—N3—C18—O4	−1.7 (3)
C7—N1—C1—N2	−34.8 (3)	C24—N3—C18—N4	13.2 (3)
C2—N1—C1—N2	176.1 (2)	C19—N3—C18—N4	179.3 (2)
C1—N1—C2—C3	−77.9 (3)	C18—N3—C19—C20'	96.2 (10)
C7—N1—C2—C3	131.4 (2)	C24—N3—C19—C20'	−96.6 (10)
C6—O2—C3—C4	−0.7 (3)	C18—N3—C19—C20	92.1 (7)
C6—O2—C3—C2	178.9 (2)	C24—N3—C19—C20	−100.7 (7)
N1—C2—C3—C4	111.0 (3)	C23—O5—C20—C21	−2.2 (19)
N1—C2—C3—O2	−68.4 (3)	C23—O5—C20—C19	−176.8 (13)
O2—C3—C4—C5	0.3 (3)	N3—C19—C20—C21	−116 (2)
C2—C3—C4—C5	−179.1 (3)	N3—C19—C20—O5	57.0 (18)
C3—C4—C5—C6	0.2 (3)	O5—C20—C21—C22	3.4 (19)
C4—C5—C6—O2	−0.7 (3)	C19—C20—C21—C22	176.8 (19)
C3—O2—C6—C5	0.9 (3)	C20—C21—C22—C23	−3.2 (13)
C1—N1—C7—C8'	−71.0 (6)	C21—C22—C23—O5	2.1 (8)
C2—N1—C7—C8'	77.9 (5)	C20—O5—C23—C22	−0.1 (12)
C1—N1—C7—C8	−69.6 (3)	C23'—O5'—C20'—C21'	−10 (3)
C2—N1—C7—C8	79.4 (3)	C23'—O5'—C20'—C19	−173 (3)
C11—O3—C8—C9	1.2 (10)	N3—C19—C20'—C21'	81 (3)
C11—O3—C8—C7	178.1 (7)	N3—C19—C20'—O5'	−118 (4)
N1—C7—C8—C9	102.4 (12)	O5'—C20'—C21'—C22'	5 (3)
N1—C7—C8—O3	−73.4 (9)	C19—C20'—C21'—C22'	171 (2)
O3—C8—C9—C10	−1.0 (11)	C20'—C21'—C22'—C23'	2 (3)
C7—C8—C9—C10	−177.0 (10)	C21'—C22'—C23'—O5'	−8 (3)
C8—C9—C10—C11	0.4 (8)	C20'—O5'—C23'—C22'	12 (3)
C9—C10—C11—O3	0.3 (6)	C18—N3—C24—C25	72.2 (3)
C8—O3—C11—C10	−1.0 (7)	C19—N3—C24—C25	−94.2 (2)
C11'—O3'—C8'—C9'	1 (2)	C28—O6—C25—C26	−0.1 (3)
C11'—O3'—C8'—C7	178.7 (14)	C28—O6—C25—C24	−179.9 (2)
N1—C7—C8'—C9'	−77 (3)	N3—C24—C25—C26	−116.6 (3)
N1—C7—C8'—O3'	106.3 (17)	N3—C24—C25—O6	63.1 (3)
O3'—C8'—C9'—C10'	−1 (2)	O6—C25—C26—C27	−0.1 (3)
C7—C8'—C9'—C10'	−178 (3)	C24—C25—C26—C27	179.6 (2)
C8'—C9'—C10'—C11'	0.1 (18)	C25—C26—C27—C28	0.3 (3)
C9'—C10'—C11'—O3'	0.6 (12)	C26—C27—C28—O6	−0.4 (3)
C8'—O3'—C11'—C10'	−1.1 (16)	C25—O6—C28—C27	0.3 (3)
C1—N2—C12—C17	−25.9 (4)	C18—N4—C29—C34	37.3 (3)
C1—N2—C12—C13	156.1 (2)	C18—N4—C29—C30	−145.6 (2)
C17—C12—C13—C14	−0.1 (4)	C34—C29—C30—C31	−1.5 (4)
N2—C12—C13—C14	178.0 (2)	N4—C29—C30—C31	−178.7 (2)
C12—C13—C14—C15	1.0 (4)	C29—C30—C31—C32	−0.5 (4)
C13—C14—C15—C16	−0.8 (4)	C30—C31—C32—C33	1.6 (4)
C14—C15—C16—C17	−0.3 (5)	C31—C32—C33—C34	−0.8 (4)
C13—C12—C17—C16	−1.0 (4)	C32—C33—C34—C29	−1.2 (4)
N2—C12—C17—C16	−179.0 (3)	C30—C29—C34—C33	2.4 (4)
C15—C16—C17—C12	1.2 (5)	N4—C29—C34—C33	179.4 (2)

### 1,1-Bis(furan-2-ylmethyl)-3-phenylurea Hydrogen-bond geometry (Å, º)

*Cg*1, *Cg*5 and *Cg*6 are the centroids of the O2/C3–C6, C12–C17 and C29–C34 rings, respectively.

**Table:**

*D*—H···*A*	*D*—H	H···*A*	*D*···*A*	*D*—H···*A*
N2—H2*N*···O1^i^	0.90	2.03	2.917 (2)	168
N4—H4*N*···O4^ii^	0.90	2.07	2.955 (2)	167
C6—H6···Cg6^iii^	0.95	2.62	3.482	150
C24—H24*B*···Cg5^iv^	0.99	2.90	3.789	149
C28—H28···Cg1^iv^	0.95	2.82	3.658	148

Symmetry codes: (i) *x*, −*y*+1/2, *z*−1/2; (ii) *x*, −*y*+3/2, *z*−1/2; (iii) −*x*, *y*+1/2, −*z*+1/2; (iv) *x*, *y*−1, *z*.
